# The use of e-consent in surgery and application to neurosurgery: a systematic review and meta-analysis

**DOI:** 10.1007/s00701-023-05776-3

**Published:** 2023-09-11

**Authors:** Asfand Baig Mirza, Abbas Khizar Khoja, Fizza Ali, Mustafa El-Sheikh, Ammal Bibi-Shahid, Jandira Trindade, Brett Rocos, Gordan Grahovac, Jonathan Bull, Alexander Montgomery, Babak Arvin, Ahmed-Ramadan Sadek

**Affiliations:** 1 Department of Neurosurgery, Queens Hospital Romford, London, UK; 2 Guy’s, King’s and St Thomas’ School of Medical Education, King’s College London, London, UK; 3 King’s College Hospital, Kings NHS Foundation Trust, Denmark Hill, London, UK; 4 Royal London Hospital, Barts Health NHS Trust, London, UK

**Keywords:** e-Consent, Electronic technologies, Informed consent, Meta-analysis, Surgery, Patient satisfaction

## Abstract

**Introduction:**

The integration of novel electronic informed consent platforms in healthcare has undergone significant growth over the last decade. Adoption of uniform, accessible, and robust electronic online consenting applications is likely to enhance the informed consent process and improve the patient experience and has the potential to reduce medico-legal ramifications of inadequate consent. A systematic review and meta-analysis was conducted to evaluate the utility of novel electronic means of informed consent in surgical patients and discuss its application to neurosurgical cohorts.

**Methods:**

A review of randomised controlled trials, non-randomised studies of health interventions, and single group pre-post design studies in accordance with the PRISMA statement. Random effects modelling was used to estimate pooled proportions of study outcomes. Patient satisfaction with the informed consent process and patients’ gain in knowledge were compared for electronic technologies versus non-electronic instruments. A sub-group analysis was conducted to compare the utility of electronic technologies in neurosurgical cohorts relative to other surgical patients in the context of patient satisfaction and knowledge gain.

**Results:**

Of 1042 screened abstracts, 63 studies were included: 44 randomised controlled trials (*n* = 4985), 4 non-randomised studies of health interventions (*n* = 387), and 15 single group pre-post design studies (*n* = 872). Meta-analysis showed that electronic technologies significantly enhanced patient satisfaction with the informed consent process (*P* < 0.00001) and patients’ gain in knowledge (*P* < 0.00001) compared to standard non-electronic practices. Sub-group analysis demonstrated that neurosurgical patient knowledge was significantly enhanced with electronic technologies when compared to other surgical patients (*P* = 0.009), but there was no difference in patient satisfaction between neurosurgical cohorts and other surgical patients with respect to electronic technologies (*P* = 0.78).

**Conclusions:**

Novel electronic technologies can enhance patient satisfaction and increase patients’ gain in knowledge of their surgical procedures. Electronic patient education tools can significantly enhance patient knowledge for neurosurgical patients. If used appropriately, these modalities can shorten and/or improve the consent discussion, streamlining the surgical process and improving satisfaction for neurosurgical patients.

**Supplementary Information:**

The online version contains supplementary material available at 10.1007/s00701-023-05776-3.

## Introduction

Providing patients with easy-to-understand information about a recommended procedure, and subsequently requesting permission from them, is the ethical and legal obligation of the modern-day clinician [22]. This protocol, defined as informed consent (IC), enables patients to exercise autonomy and participate in their medical care [32]. IC seeks to balance the doctor-patient relationship by providing a legal framework to protect both the patient from harm and the doctor from litigation [41]. It is an explicit authorisation provided by the patient to assent (consent) or dissent (refuse) a healthcare intervention offered by their physician. Empowering patients to participate in the IC process and engage in shared decision-making has become the gold standard of patient care in recent years [19, 73].

According to the UK Department of Health’s guidance on eliciting consent, a valid IC process includes not only the disclosure of information to patients but also its comprehension by them [21, 67]. However, there are concerns that the current IC process in surgical cases may be suboptimal [35, 40, 47, 53, 81]. In one study, only a third of participants demonstrated sufficient understanding of their operative risks [30]. Although suboptimal patient engagement can be a direct cause for this, it is largely due to poor disclosure of information by healthcare providers, which reduces patient understanding and hinders satisfaction [36, 44]. This leads to lower confidence in healthcare providers and affects treatment adherence, postoperative recovery, and ultimately clinical outcomes [31, 62, 93].

Due to limited time and in the interest of efficient workflow, the IC process can be truncated from a robust patient education event to a mere signature on a form [13]. Standardised information sheets can lead to some patients being neglected, especially in the presence of low health literacy or language barriers, resulting in poor comprehension and, thereby, an inadequate IC process [17]. The nature of the complex procedures comprising the practice of neurosurgery makes the challenge of achieving adequate informed consent even greater for neurosurgical patients. Further, in neurosurgery decisions regarding surgery, its risks and benefits need to be fully understood and weighed up by the patient, as it can have life-changing consequences. Neurosurgical patients can also have complex communication needs. Electronic consent offers another avenue to ensure that informed consent is established, allowing patients to take their time to fully understand and acknowledge the consent process.

Patient knowledge and patient satisfaction are the two predominant metrics used in the existing literature to assess the adequacy of the informed consent process. Both depend on the instruments utilised to disclose information [48]. The tools for information disclosure have evolved in the modern digital era. Legacy methods such as written instruments (leaflets, brochures) remain in use, but there is increasing adoption of novel electronic interventions in the form of interactive and non-interactive decision aid software, and multimedia aids such as audio recordings, videos, and compact discs (CDs) [15, 26]. Electronic tools encourage patients to listen and engage with information in their own time. The COVID-19 pandemic has been an unexpected impetus for the adoption of digital dissemination of clinically related information in health services around the world [8]. Substantial evidence has emerged suggesting that these digital modalities have enhanced the patient experience and that the value of these electronic tools can be greater in certain surgical specialties (such as neurosurgery) over others [35].

The purpose of this systematic review and meta-analysis is to report on the value of electronic technologies for the IC process in surgical patients, with a focus on neurosurgical cohorts.

## Methods

For the purposes of this systematic review, standardised definitions for the interventions and comparators were used (see Table 1).

**Table 1:** Standardised definitions

Type of intervention	Definition
Electronic technologies	Any intervention that uses electronic or digital means to carry out or support the IC process. This includes pre-recorded audio-visual media, decision-making tools, mobile/tablet applications, websites, and video/PowerPoint presentations To detect further differences in outcome, electronic technologies were subdivided into interactive and non-interactive modalities. The definitions used for these modalities for the purposes of this review are found below
Interactive	Any intervention consisting of audio-visual components that require active patient participation. These are educational tools with corresponding knowledge assessments in-built into the programme
Non-interactive	Any intervention that uses audio-visual components but does not require active patient participation. This includes pre-recorded videos and speeches
Comparator	Any intervention that does not use electronic technologies for IC. This includes standard verbal education with health professionals, written information devices such as brochures, and physical 3-dimensional models

*IC*, informed consent

### Search strategy

The study adhered to the PRISMA and AMSTAR guidelines for the design, conduct, and reporting of systematic reviews and meta-analyses [68]. A search of original publications from the following online bibliographic databases was conducted to find suitable studies: OVID MEDLINE, PubMed, Embase, Global Health, and APA PsycInfo.

The PubMed search strategy consisted of MeSH headings and keywords for “digital interventions”, “consent”, and “surgery”, along with related terms linked by the Boolean operator “AND”. This strategy was adapted for other databases (see supplemental Table A).

Two reviewers (AKK, FA) independently screened titles and abstracts of identified papers in accordance with predefined selection criteria. The second stage consisted of full-text retrieval to confirm inclusion eligibility. Reference lists of identified articles were manually searched for additional relevant publications. Studies were eligible for inclusion if they satisfied the criteria listed in Table 2.

**Table 2:** List of pre-defined eligibility criteria

PICOS and additional domains	Eligibility criteria	Notable exclusions
Population	Human patients from whom informed consent (IC) was sought for surgical procedures* Patients aged 16 years or older Patients competent to consent for themselves	Patients < 16 years Patients without capacity to consent Patients undergoing medical interventions/procedures
Intervention	Electronic technologies to enhance the IC process§ Electronic technologies could be utilised at any stage of the IC process from patient education to the signing of the consent form	–
Comparator	Standard non-electronic means for carrying out the informed consent process, e.g., verbal discussions with a doctor	–
Outcome	Satisfaction—assessed explicitly via questionnaire or verbally Objective gain in knowledge	Anxiety
Study design	Prospective design including: Randomised controlled trials Non-randomised controlled studies One-group pre-post evaluation studies	Retrospective designs Observational studies Secondary research articles Anecdotal evidence: case reports and series
Language	Written and reported in English	–
Date of publication	No restrictions	–
Availability of article	Available as a full-text report	Abstract-only reports Conference presentation extracts

^*^Patients were considered from any surgical speciality, in which invasive procedures involve either penetration of the skin or passage of an instrument through an orifice, and for which IC is routinely sought and is a legal requirement

^§^Studies were permitted if they used e-consent technologies in addition to standard practice for their intervention arm

### Registration and protocol

The review was registered on PROSPERO (International Prospective Register of Systematic Reviews) with the identification number CRD42022314812.

### Data extraction

Based on the Cochrane Review Group Data Extraction template [77], a data extraction form (DEF) was designed to collect information relevant to the study outcomes. This was tested before use. Variables collected are listed in Table 3. Missing/unclear data were noted, and no assumptions about the data were made. Three researchers (AKK, FA, and ABS) independently implemented the DEF for each study. Discrepancies were resolved through discussions with senior authors (ABM, ARS).

**Table 3:** Processes for data handling and analysis

Methods	Process
Data extraction	Collected data included: publication information (author(s), publication year, and country of study); information relating to ethics and good research practices (IC for participation in the study, report of funding and conflict of interests, and reporting of ethical approval); study and procedure characteristics (aims, design, surgical discipline, nature of invasive procedure); participant demographics (mean and SD of age, sex, ethnicity, socio-economic background, literacy, numbers of patients eligible, exclusions, and loss to follow- up); nature of electronic IC intervention (content and format); nature of comparator (content and format); information on outcomes used (description of outcome, method of assessment (outcome measure), timing of outcome measurement); and effect value for the outcomes
Data analyses	Results from studies reporting continuous data were processed in the format of mean, SD and total arm size (n). For studies that did not report SDs, the values were estimated from SE values and CIs for group means, in accordance with the guidance set out by the Cochrane Handbook of Systematic reviews of interventions. Studies with missing SE values or CIs were excluded from meta-analyses. For studies reporting categorical data, results were processed as ratios of events in the intervention arm versus events in the control arm. For meta-analysis, studies were pooled based on the format of data reported. Neurosurgical studies reported data for patient satisfaction in categorical form and patient knowledge in continuous form. Hence, this led to the following three outcomes being analysed (with the latter two having sub-groups to separate and compare non-neurosurgical studies with neurosurgical studies): - Patient satisfaction (continuous data) - Patient satisfaction (categorical data) ○ Sub-group analysis: non-neurosurgical vs. neurosurgical studies - Patient Knowledge (continuous data) ○ Sub-group analysis: non-neurosurgical vs. neurosurgical studies Continuous data were analysed with inverse variance. SMDs were selected to pool data reported in continuous format to enable comparisons between the studies as there was a diverse range of measurement scales used. Categorical data were analysed with the Mantel-Haenszel test. ORs were selected to pool data reported in categorical format between the studies. Due to the diversity of studies observed during preliminary research random effects modelling was chosen for the analysis. These studies had different: study populations; sample sizes; outcome measures (validated questionnaires vs. purpose-built instruments); electronic IC interventions; traditional non- electronic interventions; follow-up duration and research environment. The random effects model was specifically chosen to ensure that studies with larger sample sizes do not dominate the results and trivialize smaller studies. This also allowed consideration of the potential differences in the impact of electronic interventions on populations from different backgrounds (age/education level/socioeconomic status) in estimating the combined effect size, as studies differed on these grounds.

*IC* Informed consent; *SD* Standard deviation; *SE* Standard errors; *CI* Confidence intervals; *SMD* Standardised mean difference; *OR* Odds ratio

### Outcomes and outcome measures

The study outcomes, their standardised definitions, and outcome measures are listed in Table 4. As electronic IC interventions are primarily designed for patients to use outside the standard consultation with their physicians, the study outcomes were purposefully patient-centred, while other possible outcomes (e.g., uptake of procedures, complaints, and adverse clinical outcomes) were excluded.

**Table 4:** Outcomes and definitions

Type of outcome	Outcome	Definition
Primary outcome	Patient satisfaction was the primary focus of interest with the IC process as the primary outcome	To fulfil this key criterion, studies had to demonstrate that they explicitly assessed and reported patient satisfaction, and studies proxying satisfaction with metrics such as “convenience” or “ease of understanding” were not eligible. Patient satisfaction with the IC process was defined as acquiescence with the interventions used for preoperative education and information disclosure. Since this is inherently subjective, no restrictions were placed on the outcome measures
Secondary outcome	Patient Knowledge was chosen as the secondary outcome	A gain in patient knowledge was defined as an increase in patients’ understanding of their medical condition and/or knowledge related to the proposed procedure, including the benefits, risks, and alternatives. Studies meeting the eligibility criteria may have referred to this increase in understanding as “comprehension”, “knowledge”, or “recall”. In the preliminary search, seldom did studies draw a distinction between these terms. Therefore, in this review, the term patient knowledge is used to refer to the outcome that may have been alternatively phrased in the individual reports Moreover, as this is an objective outcome, only studies using forms of information-based assessments were accepted, from MCQs to true/false questions. Studies that reported a subjective gain in knowledge (e.g., self-reported measures of understanding) were excluded

*IC*, informed consent; *MCQs*, multiple-choice questions

### Risk of bias assessment

The quality of the included studies was evaluated using the National Heart, Lung, and Blood Institute (NHLBI) quality assessment tools [37]. Three reviewers (AKK, FA, ABS) independently assessed each study using the appropriate quality assessment tool and calculated a score for each study, scoring them as “good”, “fair”, or “poor” quality. “Good quality” studies had the least risk of bias, and the results were considered valid. “Fair quality” studies were susceptible to bias but not deemed sufficient to invalidate results. “Poor quality” studies had a significant risk of bias that likely invalidated the findings. If ratings differed, reviewers discussed the article to reach a consensus and, if necessary, discussed with senior authors (ABM, ARS).

### Data analyses

A table summarising relevant data for each included RCT was generated using Review Manager (Cochrane Collaboration: Version 5.4) [83]. Studies with unclear/missing data for the outcomes of interest were excluded from meta-analysis. The processes used for data analysis for the outcomes are listed in Table 3.

A random effects model was selected for the analysis due to the heterogeneity observed in the studies during the preliminary research. Studies were grouped according to the format of data recorded for patient satisfaction (primary outcome) in order to minimise heterogeneity. Forest plots were generated for each outcome to illustrate the results. Alpha values < 0.05 were considered statistically significant.

The sub-group analyses investigated whether neurosurgical cohorts experienced greater satisfaction and gained more knowledge when compared to patients of other surgical specialties, in the context of digital modalities of information disclosure. Studies reporting their findings for patient satisfaction in continuous data format were not eligible for the sub-group analyses as all neurosurgical studies reported this outcome in categorical data form.

Sensitivity analyses were also conducted to determine the potential sources of heterogeneity. To assess the robustness of the findings, each study was excluded one-by-one, and all poor-quality studies were removed. Publication bias was assessed via funnel plot analysis for outcomes with a sample size > 10.

## Results

### Study selection

**Fig. 1:**
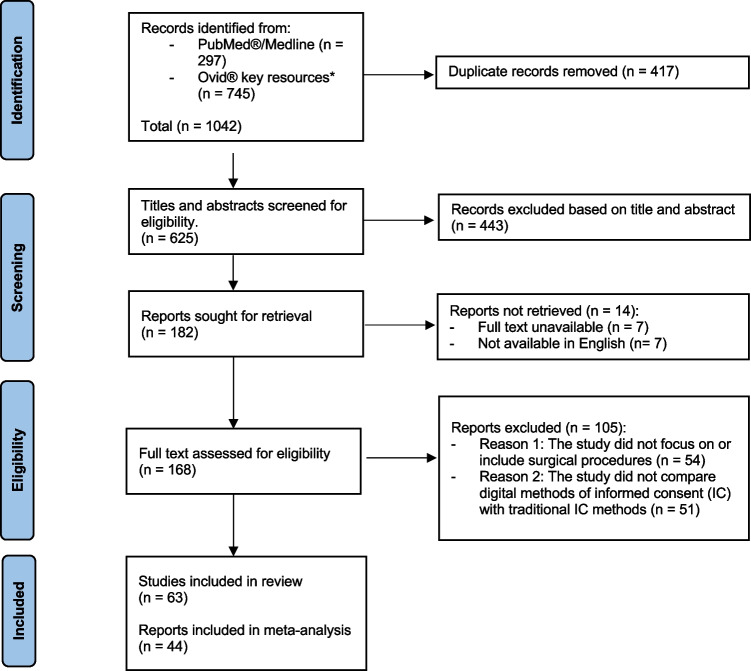
PRISMA flow diagram of search process

The search yielded 1042 studies, 625 of which were screened after duplicates were removed. Some 182 potentially eligible abstracts were identified, and a full review led to 44 RCTs, 4 non-randomised studies (NRSI), and 15 single group pre-post design (PPD) studies. Figure 1 presents a PRISMA flow chart of this process.

### Study characteristics

Tables 5, 6, 7, and 8 summarise the characteristics of the included studies (including surgical disciplines, digital modalities tested, and outcome measures used). All articles included participants with competence to consent.

**Table 5:** Findings of qualitative synthesis

Author, year, country	Study design	Outcomes	Outcome measures	Data Format	Findings
Ader et al., 1992, USA [25]	Three arm RCT	Patient satisfaction	Purpose-built Likert-type questionnaire	Continuous	Significant increase in patient satisfaction in interactive and non-interactive intervention groups compared to control group
		Patient knowledge	Purpose-built knowledge assessment	Continuous	Greater knowledge was gain in interactive videodisc intervention group compared to standard discussion group; however, less knowledge was gained compared to non-interactive videotape group
Deyo et al., 2000, USA [78]	Two arm RCT	Patient satisfaction	Purpose-built Likert-type questionnaire	Categorical	No significant difference in patient satisfaction between video group (69.5% “satisfied” or “somewhat satisfied” at 3-month follow up) and booklet group (75.1%); however, video group felt better informed
Morgan et al., 2000, Canada [63]	Two arm RCT	Patient satisfaction	Purpose-built Likert-type questionnaire	Categorical	No significant difference in patient satisfaction between interactive intervention (71%) and control (70%) groups
		Patient knowledge	Purpose built knowledge assessment	Continuous	Higher knowledge scores in intervention group (75%) compared to control (62%)
Evrard et al., 2005, England [28]	Same group PPD	Patient satisfaction	Dichotomous question	Categorical	71% of 65 patients that viewed the informative DVD were satisfied; 3% claimed it was a negative experience
			Possible answers: satisfied; not satisfied		
Kessler et al., 2005, Switzerland [47]	Same group PPD	Patient satisfaction	Purpose-built Likert-type questionnaire	Categorical	With the interactive computer programme, 63% patients were “very satisfied”, and 37% were “satisfied”, with 98% wanting to be informed by this method again
Rossi et al., 2005, USA [60]	Two arm RCT	Patient satisfaction	Purpose-built Likert-type questionnaire	Categorical	No significant difference in patient satisfaction between groups
		Patient knowledge	Purpose built knowledge assessment	Continuous	Significantly greater knowledge gain and comprehension in video group (78.5%) compared to standard verbal group (65.4%)
Sahai et al., 2006, England [83]	Same group PPD	Patient satisfaction	Client Satisfaction Questionnaire (CSQ-8)	Continuous	High mean patient satisfaction (CSQ-8) score: 29.8 (average) out of a maximum possible score of 32. A video supplement aids patient understanding
Eggers et al., 2007, Germany [10]	Two arm NRSI	Patient satisfaction	Numerical rating scale 0–7	Categorical	Greater satisfaction in intervention group
		Patient knowledge	Purpose built knowledge assessment	Continuous	Better understanding across all topics in the intervention group compared to control
Nozaki et al., 2007, Japan [69]	Same group PPD	Patient satisfaction	Dichotomous question	Categorical	After watching a DVD, 69.6% of patients were satisfied with their treatment decision, compared to 63.2% before watching the DVD which shows an increase in satisfaction following this intervention
			Possible answers: satisfied; not satisfied		
		Patient knowledge	Purpose built knowledge assessment	Continuous	The average knowledge score increased from 8.72 out of 15 to 12.4 after watching the DVD. At 3 months follow-up, this score was 10.34 which shows multimedia aids with short- and long-term comprehension
Beischer et al., 2008, USA [4]	Same group PPD	Patient satisfaction	Visual analogue scale 10 cm	Continuous	Multimedia educational material improved satisfaction and ease of understanding compared to standard discussion, with 63% of patients saying that the intervention answered their questions
		Patient knowledge	Purpose-built knowledge assessment	Categorical	The intervention improved knowledge, with average correct knowledge scores increasing from 64% (surgeon–patient discussion) to 87% (multimedia intervention). 98% felt well-informed following the intervention, compared to 68% after the standard discussion
Bollscheiler et al., 2008, Germany [26]	Two arm RCT	Patient satisfaction	Numerical rating scale 0–7	Categorical	No significant difference in patient satisfaction between multimedia intervention group (83%) and control group (80%)
Heller et al., 2008, USA [65]	Two arm RCT	Patient satisfaction	Purpose-built Likert-type questionnaire	Categorical	Intervention group was significantly more satisfied than control group with decision-making and understanding information
		Patient knowledge	Purpose built knowledge assessment	Continuous	When comparing knowledge before and after informed consent, the intervention group showed a greater mean increase of knowledge (14%) compared to control group (8%)
Migden et al., 2008, USA [53]	Two arm RCT	Patient satisfaction	Purpose-built Likert-type questionnaire	Insufficient data reported for MA	Increased patient satisfaction in the video group compared to control discussion group
		Patient knowledge	Purpose-built knowledge assessment	Insufficient data reported for MA	Increased patient comprehension and efficiency in the intervention group
Beamond et al., 2009, Australia [7]	Same group PPD	Patient satisfaction	Visual analogue scale 10 cm	Categorical	90% of patients confirmed that the intervention (multimedia) answered their questions better than a physical discussion with the surgeon
		Patient knowledge	Purpose-built knowledge assessment	Continuous	The intervention aided comprehension and knowledge, with patients answering more questions correctly after the intervention (87%) compared to before (62%)
Rigatelli et al., 2009, Italy [34]	Two arm NRSI	Patient satisfaction	Purpose-built Likert-type questionnaire	Continuous	Mean satisfaction in the multimedia intervention group was greater than control group, with scores of 2.9 ± 0.1 (intervention), compared to 1.8 ± 0.9 (control)
Tait et al., 2009, USA [88]	Two arm RCT	Patient satisfaction	Numerical rating scale 0–10	Continuous	No difference in satisfaction between groups
		Patient knowledge	Purpose-built knowledge assessment	Continuous	Intervention group showed significantly greater understanding compared to control group and understanding of risks and other options
Wilhelm et al., 2009, Germany [91]	Two arm RCT	Patient satisfaction	Purpose-built Likert-type questionnaire	Continuous	No significant difference in patient satisfaction between intervention and control group
		Patient knowledge	Purpose-built knowledge assessment	Continuous	Intervention (DVD) group showed a greater gain of knowledge compared to control group through higher scores—19.88 vs. 17.58
Armstrong et al., 2010, USA [84]	Two arm RCT	Patient satisfaction	Numerical rating scale 0–10	Continuous	Both the intervention and control group showed high levels of patient satisfaction with the informed consent
		Patient knowledge	Purpose-built MCQ knowledge assessment	Continuous	The video intervention group showed a significant increase in knowledge scores after watching the video (1.55 ± 1.71) while there was no significant change in the control group (1.12 ± 1.74)
Chantry et al., 2010, USA [12]	Two arm RCT	Patient satisfaction	Purpose-built Likert-type questionnaire	Continuous	No significant difference in satisfaction between intervention and control groups
		Patient knowledge	Purpose-built true/false knowledge assessment	Continuous	No significant difference in knowledge change between intervention and control groups, although higher education level was associated with greater knowledge
Cornoiu et al., 2010, Australia [15]	Three arm RCT	Patient satisfaction	Purpose-built Likert-type questionnaire	Continuous	Patient satisfaction was greater in the intervention group compared to verbal consent and pamphlet groups
		Patient knowledge	Purpose-built knowledge assessment	Continuous	The intervention group showed greater gain in knowledge compared to verbal and pamphlet groups, with 98% for multimedia group compared to 88% and 76% for verbal and pamphlet groups respectively
Gautschi, 2010, Switzerland [70]	Same group PPD	Patient satisfaction	Purpose-built Likert-type questionnaire	Categorical	83% were “very content” at the audio-visual intervention
Hung et al., 2011, Taiwan [39]	Two arm RCT	Patient satisfaction	Purpose-built Likert-type questionnaire	Continuous	Significant increase in satisfaction with multimedia intervention compared to control group
		Patient knowledge	Purpose built knowledge assessment	Continuous	Significant increase in comprehension with multimedia intervention compared to control group
Johnson et al., 2011, USA [64]	Three arm RCT	Patient satisfaction	Purpose-built Likert-type questionnaire	Categorical	No significant difference in satisfaction between intervention and control groups
		Patient knowledge	Purpose-built knowledge assessment	Continuous	No significant difference in gain of knowledge between the groups
Birks et al., 2012, Australia [23]	Same group PPD	Patient satisfaction	Visual analogue scale 10 cm	Continuous	Patients were most satisfied with the multimedia tool being used after patient-surgeon discussion, with 95% satisfaction at the ease of understanding and 90% satisfied at the appropriate amount of information provided
		Patient knowledge	Purpose-built knowledge assessment	Categorical	It helped increase knowledge, with patient scores at 75% before, compared to 88% after the intervention
Wollinger et al., 2012, Austria [92]	Two arm RCT	Patient satisfaction	Visual analogue scale 10 cm	Continuous	No significant difference in patient satisfaction between intervention group (89%) and control (87%) group
		Patient knowledge	Purpose-built Knowledge assessment	Continuous	Significantly greater knowledge gain with intervention group (15 correct answers out of 19) compared to control (12 correct)
Bozic et al., 2013, USA [8]	Two arm RCT	Patient satisfaction	Numerical rating scale 0–10	Continuous	Significantly greater satisfaction in intervention group compared to control group
		Patient knowledge	Purpose-built knowledge assessment	Categorical	Intervention group showed better decision-making and confidence in knowing what questions to ask during patient-surgeon discussion compared to control group. A larger portion of this group reached an informed decision within the first visit
Huber et al., 2013, Germany [41]	Two arm RCT	Patient satisfaction	Purpose-built Likert-type questionnaire	Categorical	Intervention group showed greater satisfaction compared to control group
Sherman et al., 2013, Australia [45]	Two arm RCT	Patient satisfaction	Purpose-built Likert-type questionnaire	Continuous	Intervention group reported greater satisfaction with the information provided
		Patient knowledge	Purpose-built true/false knowledge assessment	Continuous	Both intervention and control groups had similar levels of high knowledge
Batuyong et al., 2014, Australia [3]	Same group PPD	Patient satisfaction	Visual analogue scale 10 cm	Continuous	84% of patients considered the multimedia intervention and surgeon discussion to be equally as effective, with 8% claiming that the electronic intervention is better
		Patient knowledge	Purpose built knowledge assessment	Categorical	Statistically significant increase in correct answers following intervention, with scores increasing from 74 to 94%
Briggs et al., 2014, UK [55]	Same group PPD	Patient satisfaction	Visual analogue scale 10 cm	Categorical	High percentage of patients expressed satisfaction with intervention
Wang et al., 2014, USA [90]	Same group PPD	Patient satisfaction	Visual analogue scale 10 cm	Continuous	76% of patients rated the multimedia intervention as equally or better than the treating surgeon in answering questions
		Patient knowledge	Purpose-built knowledge assessment	Categorical	Significantly greater increase of correct answers about the surgery with intervention (85%) compared to verbal control discussion (61%)
Fraval et al., 2015, Australia [33]	Two arm RCT	Patient satisfaction	Client Satisfaction Questionnaire (CSQ-8)	Continuous	Statistically significant increase in patient satisfaction in intervention group compared to control group
		Patient knowledge	Deaconess informed comprehension test	Continuous	Significant increase in gain of patient knowledge in intervention group (69.25% correct answers compared to control group (47.38%)
Love et al., 2015, USA [27]	Two arm RCT	Patient satisfaction	Purpose-built Likert-type questionnaire	Continuous	No significant difference in patient satisfaction between video intervention and control groups
		Patient knowledge	Purpose-built knowledge assessment	Continuous	Intervention group showed increased knowledge scores following the video information (initial score 34.7%, final score 95.2%) compared to standard discussion (initial score 38.1%, final score 59.2%)
Park et al., 2015, South Korea [42]	Two arm RCT	Patient satisfaction	Visual analogue scale 10 cm	Continuous	Intervention group reported greater satisfaction compared to control group
		Patient knowledge	Purpose-built knowledge assessment	Continuous	No difference in understanding and knowledge between intervention and control groups
Tipotsch-Maca et al., 2015, Austria [82]	Two arm RCT	Patient satisfaction	Purpose-built Likert-type questionnaire	Categorical	Both intervention and control groups showed similarly high levels of patient satisfaction
		Patient knowledge	Purpose-built MCQ knowledge assessment	Continuous	Greater knowledge gain reported with intervention group (82% retention) compared to control group (72%)
Yin et al., 2015, USA [1]	Two arm RCT	Patient satisfaction	Purpose-built Likert-type questionnaire	Continuous	Intervention group reported greater satisfaction post-operatively with their information and education than control group
		Patient knowledge	Purpose built knowledge assessment	Categorical	Intervention group felt more informed and were significantly more likely to answer correctly to questions about their surgery, compared to control
Egekeze et al., 2016, USA [67]	Three arm RCT	Patient satisfaction	Purpose-built Likert-type questionnaire	Continuous	No significant difference in patient satisfaction between intervention and control groups
		Patient knowledge	Purpose-built MCQ knowledge assessment	Continuous	No significant improvement with the video intervention to mean comprehension scores, compared with standard verbal group and verbal + video group
Winter et al., 2016, Australia [57]	Two arm crossover RCT	Patient satisfaction	Client Satisfaction Questionnaire (CSQ-8)	Continuous	No significant difference in patient satisfaction between intervention and control groups
		Patient knowledge	Purpose-built MCQ knowledge assessment	Continuous	Significant increase in understanding (15.5% increase) and increased knowledge scores (17.8% increase) in the intervention group
Bekelis et al., 2017, USA [5]	Two arm RCT	Patient satisfaction	Adapted version of the Evaluation du Vecu de l’Anesthesie Generale (EVAN-G) questionnaire	Continuous	Virtual reality intervention led to greater patient satisfaction scores
Bowers et al., 2017, Canada [66]	Two arm RCT	Patient satisfaction	Purpose-built Likert-type questionnaire	Insufficient data reported for MA	Higher patient satisfaction for the intervention group compared to control
		Patient knowledge	Purpose-built true/false knowledge assessment	Insufficient data reported for MA	Increased comprehension scores in multimedia group
Kinman et al., 2017, USA [13]	Two arm RCT	Patient satisfaction	Purpose-built Likert-type questionnaire	Categorical	No significant difference in patient satisfaction between intervention (iPad) and control groups
		Patient knowledge	Surgical Treatment for Pelvic Organ Prolapse quiz (ST-POP)	Continuous	Significant increase in knowledge following counselling in both groups but no significant difference between groups. At 6-week follow-up, control group retained their information, but intervention group reverted to baseline
Lin et al., 2017, Taiwan [94]	Same group PPD	Patient satisfaction	Purpose-built Likert-type questionnaire	Categorical	High percentage of patients expressed satisfaction with intervention
		Patient knowledge	Purpose-built knowledge assessment	Continuous	Following video intervention, mean knowledge scores significantly increased compared to before the video, showing an increase in knowledge with the intervention
Park et al., 2017, South Korea [75]	Two arm RCT	Patient knowledge	Purpose-built knowledge assessment	Continuous	Higher mean knowledge scores in the intervention group (11.9 ± 1.3) compared to control group (10.2 ± 1.9)
Zhang et al., 2017, China [96]	Two arm RCT	Patient satisfaction	Purpose-built Likert-type questionnaire	Categorical	Intervention group (video) showed greater levels of patient satisfaction (86%) compared to control (65%)
		Patient knowledge	Purpose-built knowledge assessment	Categorical	Number of correct responses to the questionnaire remained constant for both groups 80.2% vs. 77.5% for video group and control group respectively
Baenninger et al., 2018, Switzerland [76]	Two arm RCT	Patient satisfaction	Purpose-built Likert-type questionnaire	Categorical	No significant difference in patient satisfaction between intervention and control groups
		Patient knowledge	Purpose-built true/false knowledge assessment	Non-parametric	No significant difference in patient knowledge gain between intervention and control groups
Bethune et al., 2018, Canada [22]	Two arm RCT	Patient knowledge	Purpose-built knowledge assessment	Continuous	Significant increase in understanding and increased knowledge scores as indicated by higher scores on the knowledge-based assessment in the intervention group
Marcus et al., 2018, UK [38]	Same group PPD	Patient satisfaction	Purpose-built Likert-type questionnaire	Categorical	High percentage of patients expressed satisfaction with intervention
		Patient knowledge	Purpose-built knowledge assessment	Continuous	The median questionnaire scores were significantly greater after the introduction of the website (14 of 15 vs. 12 of 15; P = 0.002)
Shivaprasad et al., 2018, India [81]	Three arm RCT	Patient satisfaction	Purpose-built Likert-type questionnaire	Continuous	Intervention group (video) showed significantly higher satisfaction levels than control group
Lin et al., 2018, Taiwan [95]	Two arm RCT	Patient satisfaction	Purpose-built Likert-type questionnaire	Categorical	Significant increase in patient satisfaction with the video intervention compared to control group
		Patient knowledge	Purpose-built knowledge assessment	Continuous	Higher mean knowledge scores in the intervention group (72.57 ± 16.21) compared to control group (61.67 ± 18.39)
Pallett et al., 2018, USA [74]	Two arm RCT	Patient satisfaction	Client Satisfaction Questionnaire (CSQ-8)	Continuous	No significant difference in patient satisfaction between intervention and control groups
		Patient knowledge	Purpose-built knowledge assessment	Continuous	Subjects in the video arm scored nearly 10% higher on post counselling questionnaires than the control arm
Vo et al., 2018, USA [86]	Two arm RCT	Patient satisfaction	Purpose-built Likert-type questionnaire	Continuous	No significant difference in patient satisfaction between intervention and control groups
		Patient knowledge	Purpose-built knowledge assessment	Continuous	No significant difference in patient gain in knowledge between intervention and control groups
Book et al., 2019, Germany [29]	Two arm RCT	Patient satisfaction	Purpose-built Likert-type questionnaire	Continuous	No significant difference in patient satisfaction between intervention and control groups
		Patient knowledge	Purpose-built knowledge assessment	Continuous	Intervention group showed higher overall knowledge score compared to control group
Delcambre et al., 2019, USA [56]	Two arm RCT	Patient satisfaction	Purpose-built Likert-type questionnaire	Categorical	No significant difference in patient satisfaction between intervention and control groups
		Patient knowledge	Purpose-built knowledge assessment	Continuous	Improved understanding and knowledge of the surgical procedure in the intervention group
Laskin et al., 2019, USA [19]	Same group PPD	Patient satisfaction	Purpose-built Likert-type questionnaire	Continuous	Most patients preferred the video informative intervention (62%) to the standard oral presentation (38%)
Lee et al., 2019, Republic of Korea [75]	Two arm NRSI	Patient satisfaction	Numerical rating scale 0–10	Continuous	Intervention group showed greater satisfaction levels compared to control group
			Purpose-built knowledge assessment	Categorical	
Mawhinney et al., 2019, UK [35]	Same group PPD	Patient satisfaction	Client Satisfaction Questionnaire (CSQ-8)	Continuous	High patient satisfaction with the intervention, with mean patient satisfaction (CSQ-8) score of 30.2 out of a maximum of 32
Truong et al., 2019, Australia [21]	Two arm RCT	Patient satisfaction	Visual analogue scale?	Categorical	No significant difference in patient satisfaction between intervention and control groups
		Patient knowledge	Purpose-built knowledge assessment	Continuous	Both the intervention and control group showed significant increases in knowledge scores following counselling with no significant difference between the groups
Miao et al., 2020, Australia [59]	Two arm RCT	Patient satisfaction	Purpose-built Likert-type questionnaire	Continuous	Significant increase in patient satisfaction in intervention group compared to control group. 78.4% would prefer to view the video before surgeon consent discussion
		Patient knowledge	Purpose-built knowledge assessment	Continuous	Significant increase in knowledge gain in intervention group compared to control group
West et al., 2020, USA [49]	Two arm NRSI	Patient satisfaction	Purpose-built Likert-type questionnaire	Continuous	No significant difference in patient satisfaction between intervention and control groups, however high levels in both groups
		Patient knowledge	Purpose-built knowledge assessment	Continuous	Significant improvement in patient understanding following electronic intervention
Moore et al., 2021, South Africa [61]	Two arm crossover RCT	Patient satisfaction	Client Satisfaction Questionnaire (CSQ-8)	Continuous	Significant increase in patient satisfaction in the intervention group compared to control group
Ruiss et al., 2021, Austria [58]	Two arm RCT	Patient satisfaction	Visual analogue scale 10 cm	Continuous	Significant increase in satisfaction scores in PC intervention group (75%) compared to non-PC control group (58%)
		Patient knowledge	Purpose-built knowledge assessment	Continuous	No significant difference in patient gain of knowledge between intervention and control groups
Jeney et al., 2022, USA [80]	Two arm RCT	Patient satisfaction	Purpose-built Likert-type questionnaire	Non-parametric	No significant difference in patient satisfaction between intervention and control groups
		Patient knowledge	Purpose-built knowledge assessment	Continuous	Significant increase in knowledge gain in intervention group (+ 8.5) compared to control group (+ 2.0)
Zevin et al., 2022, Canada [95]	Two arm RCT	Patient satisfaction	Client Satisfaction Questionnaire (CSQ-8)	Continuous	No significant difference in patient satisfaction between intervention and control groups
		Patient knowledge	Purpose-built knowledge assessment	Continuous	Significant increase in knowledge gain in intervention group (85%) compared to control group (78.7%)

*MA*, meta-analysis; *RCT*, randomised control trials; *NSRI*, non-randomised studies of healthcare interventions; *PPD*, pre-post design

**Table 6:** Summary of included single group pre-post design studies, including their risk of bias assessment

Author, year, country	Surgical discipline	Electronic intervention type	Intervention modality	Quality assessment
Evrard et al., 2005, England [29]	Surgical oncology	Interactive multimedia programme	Pre-recorded DVD	Poor—high risk of bias
Kessler et al., 2005, Switzerland [45]	General surgery	Interactive multimedia programme	Menu-driven computer program	Fair—low risk of bias
Sahai et al., 2006, England [43]	Urology	Interactive multimedia programme	Pre-recorded video	Fair—low risk of bias
Nozaki et al., 2007, Japan [66]	Neurosurgery	Non-interactive multimedia programme	Pre-recorded DVD	Fair—low risk of bias
Beischer et al., 2008, USA [5]	Foot & ankle surgery	Interactive Multimedia programme	Menu-driven computer program	Poor—high risk of bias
Beamond et al., 2009, Australia [4]	Foot & ankle surgery	Interactive multimedia programme	Menu-driven computer program	Fair—low risk of bias
Gautschi, 2010, Switzerland [34]	Neurosurgery	Interactive multimedia programme	Pre-recorded video	Poor—high risk of bias
Birks et al., 2012, Australia	Orthopaedics	Interactive multimedia programme	Menu-driven computer program	Fair—low risk of bias
Batuyong et al., 2014, Australia [3]	Orthopaedics	Interactive multimedia programme	Menu-driven computer program	Fair—low risk of bias
Briggs et al., 2014, UK [14]	Neurosurgery	Interactive multimedia programme	Digital (e-book) modules via iPad	Fair—low risk of bias
Wang et al., 2014, USA [88]	Foot & ankle surgery	Interactive multimedia programme	Menu-driven computer program	Fair—low risk of bias
Lin et al., 2017, Taiwan [54]	Trauma & orthopaedics	Non-interactive multimedia programme	Pre-recorded video	Fair—low risk of bias
Marcus et al., UK	Neurosurgery	Interactive multimedia programme	Website with video animations	Fair—low risk of bias
Laskin et al., 2019, USA	Dental surgery	Non-interactive multimedia programme	Pre-recorded video	Fair—low risk of bias
Mawhinney et al., 2019, UK [57]	Neurosurgery	Non-interactive multimedia programme	Pre-recorded video	Fair—low risk of bias

*PPD*, pre-post design; *DVD*, digital-video disc

**Table 7:** Summary of included non-randomised studies including risk of bias

Author, year, country	Study design	Surgical discipline	Electronic intervention type	Intervention modality	Comparator	Quality assessment
Eggers et al., 2007, Germany [28]	Two arm NRSI	General surgery	Interactive multimedia programme	Interactive storybook	Standard verbal information and printed materials provided pre-operation	Fair—low risk of bias
Rigatelli et al., 2009, Italy [74]	Two arm NRSI	Cardiothoracic	Non-interactive multimedia programme	Pre-recorded video	Standard verbal information and printed materials provided pre-operation	Poor—high risk of bias
Lee et al., 2019, South Korea	Two arm NRSI	Neurosurgery	Non-interactive multimedia programme	Pre-recorded videos	Standard verbal information and printed materials provided pre-operation	Poor—high risk of bias
West et al., 2020, USA [89]	Two arm NRSI	Dermatology	Non-interactive multimedia programme	Pre-recorded videos	Standard verbal information and printed materials provided pre-operation	Fair—low risk of bias

**Table 8:** Summary of included randomised controlled trials including risk of bias

Author, year, country	Study design	Surgical discipline	Electronic intervention type	Intervention Modality	Comparator	Quality assessment
Ader et al., 1992, USA [25]	Three arm RCT	Oral & maxillofacial	Interactive multimedia programme Non-interactive multimedia programme	Menu-driven videodisc	Standard verbal information and printed materials provided pre-operation	Poor—high risk of bias
Deyo et al., 2000, USA [23]	Two arm RCT	Neurosurgery	Interactive multimedia programme	Menu-driven videodisc	Printed materials only	Fair—low risk of bias
Morgan et al., 2000, Canada [63]	Two arm RCT	Vascular surgery	Interactive multimedia programme	Menu-driven videodisc	Standard verbal information and printed materials provided pre-operation	Fair—low risk of bias
Rossi et al., 2005, USA [75]	Two arm RCT	Orthopaedics	Non-interactive multimedia programme	Pre-recorded video	Standard verbal information and printed materials provided pre-operation	Fair—low risk of bias
Bollscheiler et al., 2008, Germany	Two arm RCT	General surgery	Interactive multimedia programme	Menu-driven videodisc	Standard verbal information and printed materials provided pre-operation	Fair—low risk of bias
Heller et al., 2008, USA [65]	Two arm RCT	Plastic and reconstructive surgery	Interactive multimedia programme	Menu-driven computer program	Standard verbal information and printed materials provided pre-operation	Poor—high risk of bias
Migden et al., 2008, USA [60]	Two arm RCT	Dermatological surgery	Interactive multimedia programme	Menu-driven computer program	Standard verbal information and printed materials provided pre-operation	Poor—high risk of bias
Tait et al., 2009, USA [82]	Two arm RCT	Vascular surgery	Interactive multimedia programme	Menu-driven computer program	Standard verbal information and printed materials provided pre-operation	Fair—low risk of bias
Wilhelm et al., 2009, Germany [90]	Two arm RCT	General surgery	Non-interactive multimedia programme	Pre-recorded video	Standard verbal information and printed materials provided pre-operation	Fair—low risk of bias
Armstrong et al., 2010, USA [24]	Two arm RCT	Dermatological surgery	Non-interactive multimedia programme	Pre-recorded video	Standard verbal information and printed materials provided pre-operation	Good—very low risk of bias
Chantry et al., 2010, USA [16]	Two arm RCT	Paediatric surgery	Non-interactive multimedia programme	Pre-recorded video	Unrelated sham video in addition to standard information provided pre-operation	Fair—low risk of bias
Cornoiu et al., 2011, Australia [18]	Three arm RCT	Orthopaedics	Interactive multimedia programme	Menu-driven computer program	Control 1: standard verbal information and printed materials provided pre-operation Control 2: written pamphlets only group	Fair—low risk of bias
Hung et al., 2011, Taiwan [56]	Two arm RCT	Orthopaedics	Interactive multimedia programme	Menu-driven computer program	Standard verbal information and printed materials provided pre-operation	Fair—low risk of bias
Johnson et al., 2011, USA [42]	Three arm RCT	Orthopaedics	Non-interactive multimedia programme	Pre-recorded video	Standard verbal information and printed materials provided pre-operation	Fair—low risk of bias
Wollinger et al., 2012, Austria [92]	Two arm RCT	Ophthalmology	Interactive multimedia programme	Menu-driven computer program	Standard verbal information and printed materials provided pre-operation	Fair—low risk of bias
Bozic et al., 2013, USA [12]	Two arm RCT	Orthopaedics	Interactive multimedia programme	Menu-driven videodisc	Standard verbal information and printed materials provided pre-operation	Fair—low risk of bias
Huber et al., 2013, Germany [38]	Two arm RCT	Urology	Interactive multimedia programme	Menu-driven computer program	Standard verbal information and printed materials provided pre-operation	Good—very low risk of bias
Sherman et al., 2013, Australia [79]	Two arm RCT	Plastics and reconstruction	Interactive multimedia programme	Menu-driven computer program	Standard verbal information and printed materials provided pre-operation	Fair—low risk of bias
Fraval et al., 2015, Australia [33]	Two arm RCT	Orthopaedics	Interactive patient information website	Patient information website	Standard verbal information and printed materials provided pre-operation	Fair—low risk of bias
Park et al., 2015, South Korea [71]	Two arm RCT	Urology	Non-interactive multimedia programme	PowerPoint® presentation	Standard verbal information and printed materials provided pre-operation	Fair—low risk of bias
Tipotsch-Maca et al., 2015, Austria	Two arm RCT	Ophthalmology	Non-interactive multimedia programme	PowerPoint® presentation	Standard verbal information and printed materials provided pre-operation	Fair—low risk of bias
Yin et al., 2015, USA [94]	Two arm RCT	Orthopaedics	Interactive multimedia programme	Menu-driven computer program	Standard verbal information and printed materials provided pre-operation	Fair—low risk of bias
Egekeze et al., 2016, USA [27]	Three arm RCT	Orthopaedics	Non-interactive multimedia programme	Pre-recorded video	Standard verbal information and printed materials provided pre-operation	Good—very low risk of bias
Love et al., 2016, USA [55]	Two arm RCT	Dermatology	Non-interactive multimedia programme	Pre-recorded video	Standard verbal information and printed materials provided pre-operation	Fair—low risk of bias
Winter et al., 2016, Australia [91]	Two arm crossover RCT	Urology	Non-interactive multimedia programme	Pre-recorded video	Standard verbal information and printed materials provided pre-operation	Fair—low risk of bias
Bekelis et al., 2017, USA [6]	Two arm RCT	Neurosurgery	Interactive multimedia virtual reality (VR) programme	Virtual reality programme	Standard verbal information and printed materials provided pre-operation	Good—very low risk of bias
Bowers et al., 2017, Canada [11]	Two arm RCT	Vascular surgery	Non-interactive multimedia programme	Pre-recorded video	Standard verbal information and printed materials provided pre-operation	Fair—low risk of bias
Kinman et al., 2017, USA	Two arm RCT	Obstetrics & gynaecology	Interactive multimedia programme	Menu-driven iPad application	Standard verbal information and printed materials provided pre-operation	Fair—low risk of bias
Park et al., 2017, South Korea [70]	Two arm RCT	Neurosurgery	Interactive multimedia programme	Menu-driven computer program	Standard verbal information and printed materials provided pre-operation	Fair—low risk of bias
Zhang et al., 2017, China [96]	Two arm RCT	Ophthalmology	Non-interactive multimedia programme	Pre-recorded video	Standard verbal information and printed materials provided pre-operation	Good—very low risk of bias
Baenninger et al., 2018, Switzerland [1]	Two arm RCT	Ophthalmology	Non-interactive multimedia programme	Pre-recorded video	Standard verbal information and printed materials provided pre-operation	Fair—low risk of bias
Bethune et al., 2018, Canada [7]	Two arm RCT	Neurosurgery	Interactive multimedia programme	Menu-driven e-book iPad application	Standard verbal information and printed materials provided pre-operation	Fair—low risk of bias
Lin et al., 2018, Taiwan [52]	Two arm RCT	Trauma & orthopaedics	Non-interactive multimedia programme	Pre-recorded video	Standard verbal information and printed materials provided pre-operation	Fair—low risk of bias
Pallett et al., 2018, USA [69]	Two arm RCT	Obstetrics & gynaecology	Non-interactive multimedia programme	Pre-recorded video	Standard verbal information and printed materials provided pre-operation	Fair—low risk of bias
Shivaprasad et al., 2018, India	Three arm RCT	General surgery	Non-interactive multimedia programme	Pre-recorded video	2D and 3D diagrams in addition to Standard verbal information and printed materials provided pre-operation	Fair—low risk of bias
Vo et al., 2018, USA [87]	Two arm RCT	Ophthalmology	Non-interactive multimedia programme	Pre-recorded video	Standard verbal information and printed materials provided pre-operation	Fair—low risk of bias
Book et al., 2019, Germany	Two arm RCT	Paediatric	Non-interactive multimedia programme	Pre-recorded video	Standard verbal information and printed materials provided pre-operation	Fair—low risk of bias
Delcambre et al., 2019, USA	Two arm RCT	Dermatology	Non-interactive multimedia programme	Pre-recorded video	Standard verbal information and printed materials provided pre-operation	Fair—low risk of bias
Miao et al., 2020, Australia [59]	Two arm RCT	Dermatology	Non-interactive multimedia programme	Pre-recorded video	Standard verbal information and printed materials provided pre-operation	Fair—low risk of bias
Truong et al., 2020, Australia [86]	Two arm RCT	Obstetrics & gynaecology	Non-interactive multimedia programme	Pre-recorded video	Printed materials only	Fair—low risk of bias
Moore et al., 2021, South Africa [61]	Two arm crossover RCT	Urology	Non-interactive multimedia programme	Pre-recorded video	Standard verbal information and printed materials provided pre-operation	Fair—low risk of bias
Ruiss et al., 2021, Austria [76]	Two arm RCT	Ophthalmology	Non-interactive multimedia programme	Pre-recorded video	Unrelated sham video in addition to standard information provided pre-operation	Fair—low risk of bias
Jeney et al., 2022, USA	Two arm RCT	Obstetrics & gynaecology	Non-interactive multimedia programme	Pre-recorded video	Standard verbal information and printed materials provided pre-operation	Fair—low risk of bias
Zevin et al., 2022, Canada [95]	Two arm RCT	General surgery	Interactive multimedia programme	Menu-driven computer program	Standard verbal information and printed materials provided pre-operation	Fair—low risk of bias

*RCT*, randomised controlled trial

### RCTs

A total of 4985 patients were randomised across 44 RCTs. Individual studies ranged from 11 to 393 participants. Forty-two articles [1, 6, 7, 9, 11, 12, 18, 20, 23–25, 27, 33, 39, 40, 42, 46, 52, 55, 58, 61–63, 65, 72, 75, 76, 78, 79, 82, 85, 86, 90, 91, 94, 95] included participants who could consent for themselves. The rest (*n* = 2) [10, 16] included parents who consented on behalf of their children who were undergoing surgical procedures. Most studies used a two-arm parallel RCT design (*n* = 39) [1, 6, 7, 9, 13, 14, 16, 20, 23, 24, 33, 39, 40, 46, 52, 55, 59, 62, 63, 65, 72, 75, 76, 78, 79, 82, 85, 86, 90, 91, 94, 95] to compare a purpose-built digital intervention for the IC process with control groups receiving traditional non-electronic IC practice. Two of these 39 RCTs implemented a cross-over design where participants served as their own controls [61, 91]. The remaining five studies were three-arm parallel RCTs [18, 25, 27, 42, 58], four of which compared their digital intervention against two different forms of non-electronic traditional means, namely standard verbal discussions, written brochures, and simple 3D diagrams/models [18, 27, 42, 58]. The remaining three-arm study compared two different electronic technologies for IC, namely an interactive video program and a non-interactive pre-recorded video, against standard verbal discussion.

### Non-randomised studies of healthcare interventions (NRSIs)

A total of 387 patients participated in the included 4 NRSIs. Individual studies ranged from 40 to 187 participants.

### One group pre-post design (PPD) studies

A total of 872 patients participated in the 15 PPD studies. Individual studies ranged from 10 to 278 participants.

### Interventions

The electronic interventions used in the studies included pre-recorded videos (*n* = 30), menu-driven videodiscs (*n* = 4), computer programs (*n* = 25), iPad applications (*n* = 2), PowerPoint® presentations (*n* = 2), patient information websites (*n* = 2), virtual reality (VR) (*n* = 1), and an interactive storybook (*n* = 1). The interventions were divided into interactive (*n* = 31) and non-interactive (*n* = 32). Interactive electronic interventions were predominantly self-paced computer programs with high-quality videos and animated graphics. Some studies used more advanced software, such as Tait et al., who used advanced 2-dimensional (2D) and 3-dimensional (3D) graphic technology to simulate various physiologic functions to aid patient-specific education on their conditions and planned procedures. Heller et al. integrated patient testimonials with before-and-after photos, while Wollinger et al. used a program developed on a touchscreen monitor with headphones, with animated 3D figures and modules to divide the information provided.

Non-interactive electronic interventions included pre-recorded videos and PowerPoint presentations, which did not require active patient participation, but still included images, animations, and authentic surgical footage.

Consenting modalities used in the control arms included the standard verbal discussion with the surgeon and written materials (*n* = 59), but in three studies, written information was provided alone. One study used physical models during consultation with the surgeon to facilitate discussion.

### Risk of bias assessment

Tables 6, 7, and 8 illustrate the results of the risk of bias assessment.

#### RCTs

Of the included RCTs, 30 (68.2%) had adequate random-sequence generation, 16 (36.4%) had satisfactory allocation concealment, four (9.1%) double-blinded, 16 (36.4%) blinded outcome assessors, 38 (86.4%) had low risk of attrition bias, and 20 (45.5%) reported sample size calculations and recruited sufficiently to detect differences in outcomes. Many items (*n* = 57, 9.7%) were recorded as unclear due to insufficient information reported.

Overall, studies were divided into good (*n* = 5) [6, 24, 27, 38, 96], fair (*n* = 36) [1, 9, 13, 14, 16, 18, 20, 23, 33, 39, 42, 46, 52, 55, 58, 59, 61, 63, 69, 71, 75, 76, 78, 79, 82, 85, 86, 90, 91, 94, 95], and poor (*n* = 3) [25, 60, 65] quality.

#### Same group PPD studies

Of the 15 PPD studies, ten (66.7%) reported pre-defined eligibility criteria and recruited representative participants from a defined clinical population. Only three (20.0%) enrolled participants met the predetermined eligibility criteria, while seven (46.7%) reported a sample size calculation and recruited the required number of participants to give confidence in the results. Only one study [67] blinded outcome assessors. All 15 studies had an attrition rate of < 20%, but five (33.3%) did not consider lost-to-follow-up in their analysis. Overall, studies were divided into those with fair (*n* = 12) [2, 6, 43, 45, 49, 54, 57, 66, 88] and poor (*n* = 3) [5, 29, 34] quality.

### Synthesis of results

Findings of the qualitative and quantitative syntheses are summarised in Tables 5 and 9, respectively. Sixty-one of 63 included studies reported the primary outcome, patient satisfaction. Forty-eight publications (76.1%) measured the secondary outcome, patient knowledge gain [1, 2, 4, 5, 9, 13, 14, 16, 18, 20, 24, 25, 27, 28, 33, 34, 39, 42, 46, 52, 54, 55, 59, 62, 63, 65, 66, 71, 75, 76, 78, 79, 82, 85–91, 94, 95]. Of the 109 outcomes measured across the 63 publications, only 10 (9.2%) were assessed using validated instruments.

**Table 9:** Summary of outcomes and meta-analysis

Outcome	Data type	SMDs or ORs	95% CIs	*I*^2^ value (%)	Direction of finding
Patient satisfaction with IC process	Continuous	SMD 0.53	0.27–0.80	88	Favours electronic IC arm
Patient satisfaction with IC process	Categorical	OR 1.90	1.58–2.29	0	Favours electronic IC arm
Objective gain in knowledge	Continuous	SMD 0.63	0.50–0.77	75	Favours electronic IC arm

*SMD*, standardised mean difference; *OR*, odds ratio; *CI*, confidence interval

#### Primary outcome: patient satisfaction with the IC process

Questionnaires used to measure patient satisfaction included validated instruments, such as the Client Satisfaction Questionnaire-8 (CSQ-8) [81] (*n* = 7) [33, 43, 57, 61, 69, 91, 95]. This is an instrument used to assess patient satisfaction in most clinical settings, in which patients are asked to rate the services received from their healthcare provider. It contains 8 items relating to patient satisfaction, with a maximum of 4 points per item, leading to a maximum score of 32. One study [6] used a scale adapted from the validated 26-item Evaluation du Vecu de l’Anesthesie Generale (EVAN-G) [84].

On the other hand, most studies utilised purpose-built measures, such as Likert scales (*n* = 37) [1, 6, 7, 10, 11, 16, 18, 20, 23, 25, 27, 34, 39, 40, 42, 45, 46, 49, 51, 54, 55, 58, 61, 63, 65, 75, 78, 79, 85, 87, 89, 90, 94, 96], 10-cm visual analogue scales (*n* = 9) [2, 6, 7, 71, 76, 86, 88, 92], and linear numerical rating scales (*n* = 6) [9, 12, 24, 28, 51, 82]. The 10-cm visual analogue scales instructed patients to put a dash on a line that was then quantified and tallied by computer software.

In contrast, two studies used a single dichotomous question aimed at overall satisfaction: “Are you satisfied with the IC process?” [29, 66].

Assessments of patient satisfaction were carried out at different times, ranging from before the operation to 6 weeks, 3 months, and 1 year after the operation. The number of items in the questionnaires to assess patient satisfaction ranged from one to 18, with individual items relating to various aspects of electronic interventions, such as appeal, comprehensibility, convenience, usefulness, information quantity, and duration of intervention.

#### Secondary outcome: patient knowledge

Outcome measures for patient knowledge were predominantly comprehension-based assessments that evaluated information retention and recall. These ranged in composition from 5 to 28 questions and included various formats, such as multiple-choice questions (MCQs), true/false items, and dichotomous (yes/no) questions. They were conducted before and after the IC intervention at various time points, ranging from pre-procedure (baseline) to 3 months and a year after the operation.

#### Results of quantitative synthesis

**Fig. 2:**
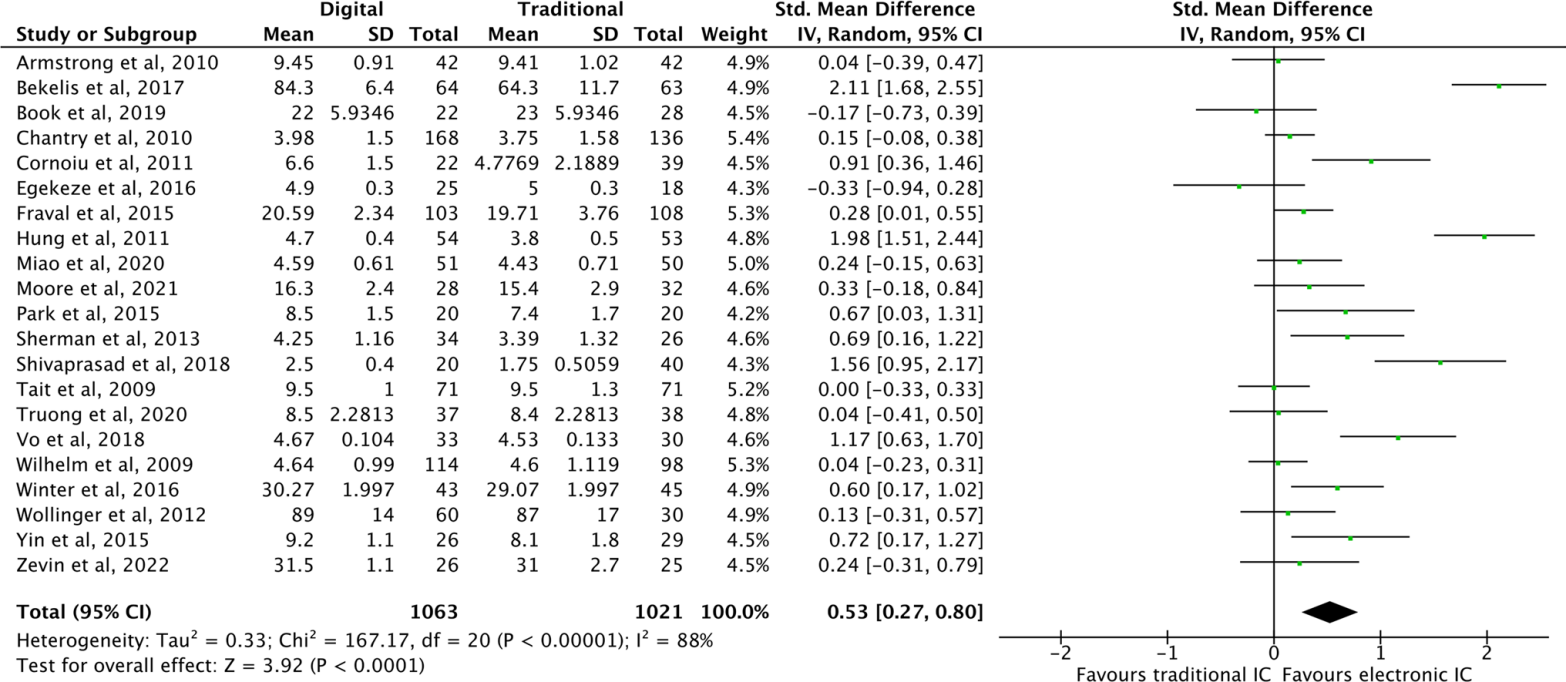
Forest plot of studies reporting patient satisfaction (continuous data)

For patient satisfaction with the IC process (continuous data), meta-analysis showed that electronic IC technologies significantly improved patient satisfaction compared to standard IC practices (*P* < 0.0001) (Fig. 2).

**Fig. 3:**
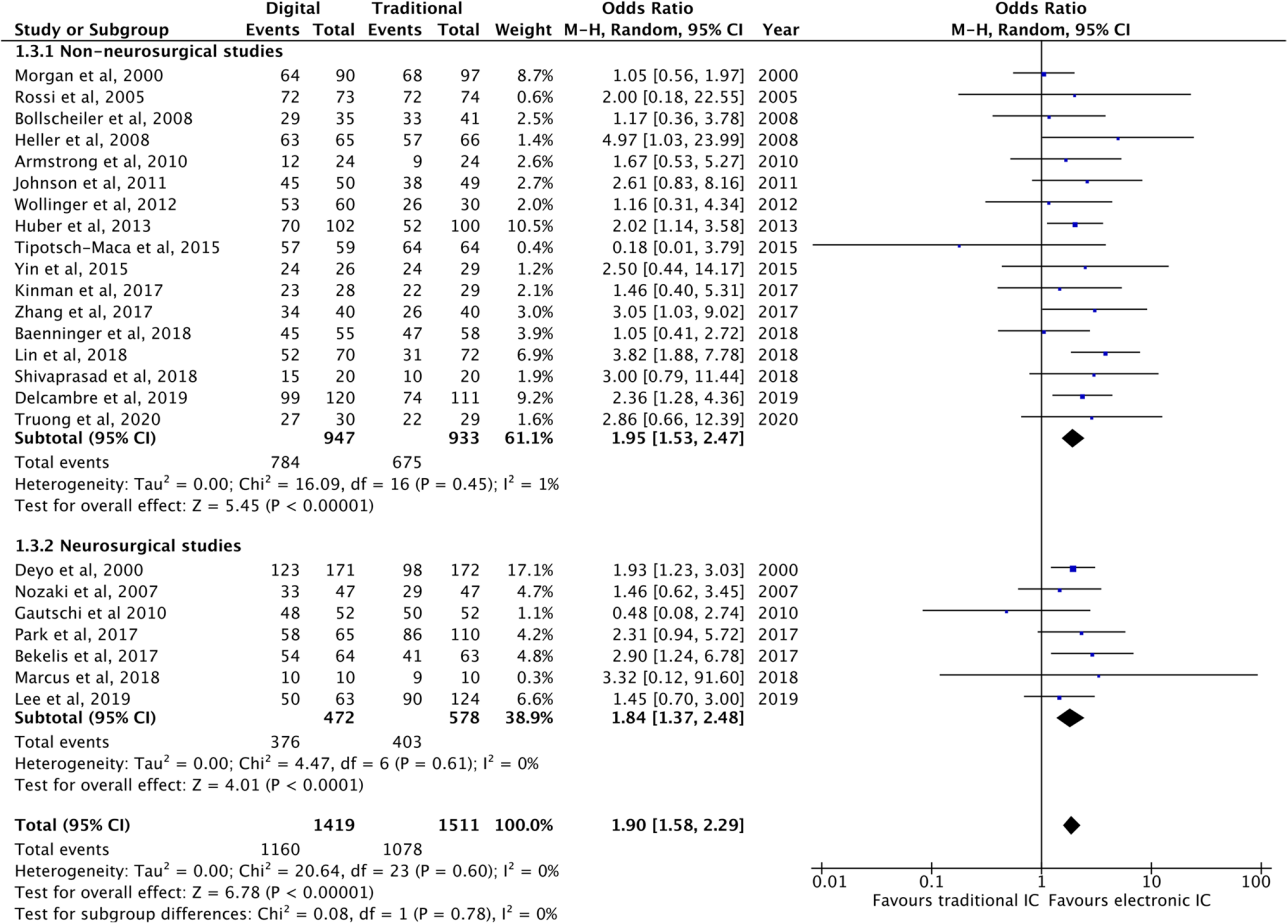
Forest plot of studies reporting patient satisfaction (categorical data) and sub-group comparison

Meta-analysis of patient satisfaction with the IC process (categorical data) demonstrated that electronic IC technologies resulted in significantly greater satisfaction compared to standard IC practices (OR 1.90; *P* < 0.00001) (Fig. 3). In other words, surgical patients undergoing the IC process via electronic tools were 1.9 times more likely to be satisfied with their medical procedure and care than those that received the traditional IC process.

The studies that reported categorical data for patient satisfaction were also significantly more homogeneous in methodology as demonstrated by an *I*^2^ value of 0%. Sub-group analysis demonstrated that this significant enhancement of patient satisfaction prevailed for neurosurgical studies (OR 1.84; *P* < 0.0001) and for non-neurosurgical studies (OR 1.95; *P* < 0.00001) (Fig. 3). There were no statistically significant differences between the sub-groups (*P* = 0.78).

**Fig. 4:**
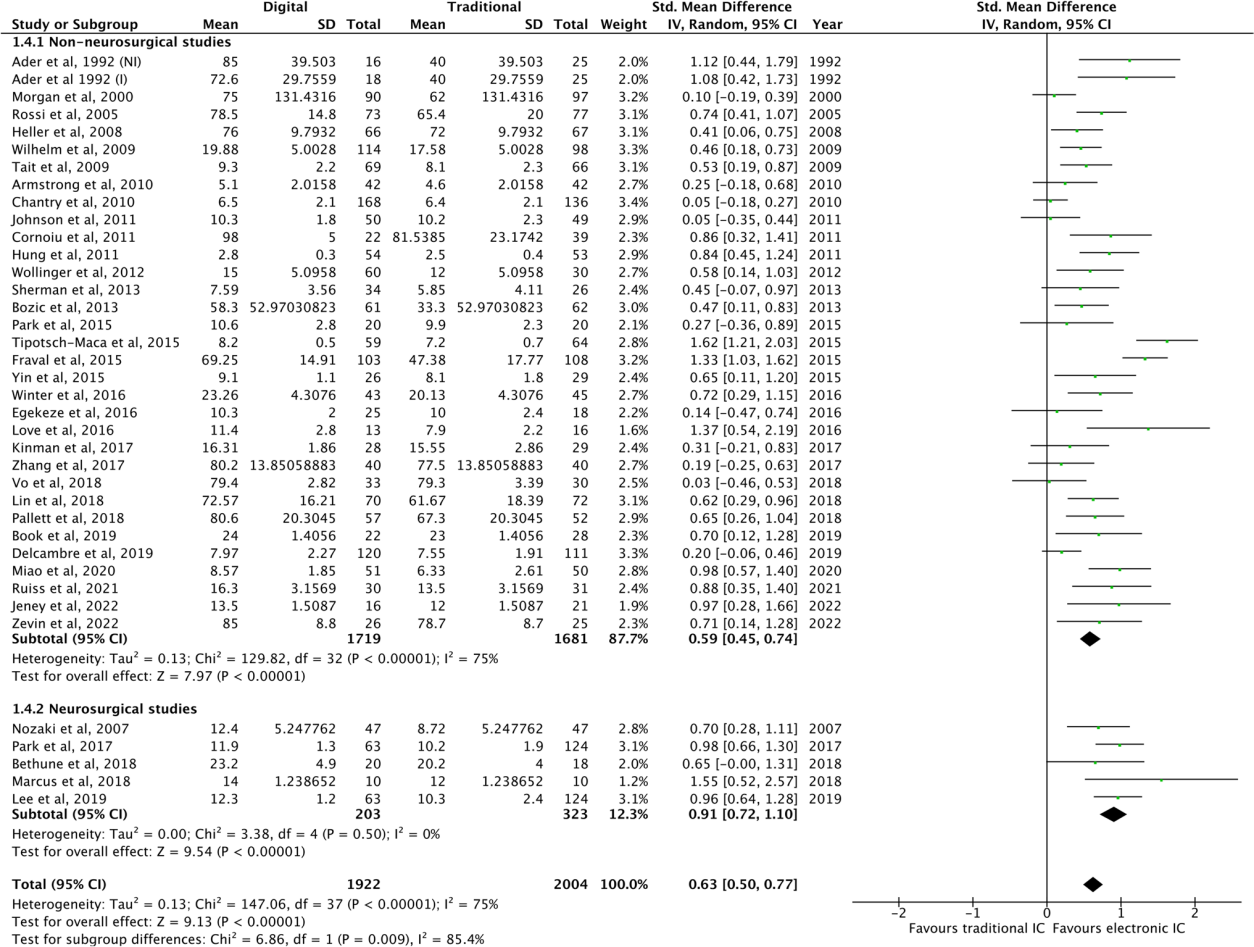
Forest plot of studies reporting objective gain in knowledge (continuous data) and sub-group comparison

For patient knowledge gain (continuous data), meta-analysis demonstrated that electronic IC technologies were significantly superior to traditional IC practices (SMD 0.63; *P* < 0.00001) (Fig. 4). Sub-group analysis showed that this significant improvement in patient knowledge through electronic technologies prevailed for neurosurgical studies (SMD 0.91; *P* < 0.00001) and for non-neurosurgical studies (SMD 0.59; *P* < 0.00001) (Fig. 4). Further, there was a statistically significant difference between neurosurgical and non-neurosurgical studies (*P* = 0.009), indicating that neurosurgical patients benefitted more in knowledge gain through digital informed consent modalities when compared to non-neurosurgical patients.

#### Results of qualitative synthesis

For patient satisfaction with the IC process, all studies (100%) reported improved satisfaction after electronic IC intervention (Table 5). Twenty-two out of 46 studies with control groups (47.8%) showed significantly greater satisfaction in electronic IC intervention groups than in controls. None of the studies showed greater satisfaction in control groups than in intervention groups. Of 45 studies that reported knowledge gain, 38 studies (84.4%) reported improved understanding following IC interventions. Thirty-four of these 38 studies (89.5%) reported significantly greater understanding after electronic IC intervention. Of the 47 studies with control groups, 39 (83.0%) reported patient knowledge gain as an outcome. Of these 39 studies, 18 (46.2%) reported significantly higher test scores in electronic IC intervention groups than in control.

#### Results of publication bias and sensitivity analyses

Funnel plot analysis did not demonstrate the presence of publication bias, with the spread of effect narrowing as the size of the study increased. Sensitivity analyses were performed for each outcome by removing each study one by one, and then excluding poor-quality studies. The removal of any of the studies did not have a disruptive effect on the findings, indicating that the pooled results were robust.

## Discussion

With the progressive digitalization of healthcare, traditional means of consent have slowly been replaced by multimedia-aided consent, which can include informative videos, interactive animations, and consent-specific platforms. The results show that this ‘e-consent’ significantly improves patient satisfaction with the IC process and patients’ knowledge gain compared to standard consent methods (e.g., patient-surgeon discussion, brochures). These outcomes are interlinked, as increased knowledge gained during the IC process and greater general understanding of the procedure, risks, and treatment options leads to greater satisfaction. Increased understanding can also reduce preoperative anxiety, which directly influences satisfaction. Although these are qualitative factors, they were assessed on numerical scales, which enabled quantitative analysis of the outcomes.

The aim of this study was to investigate how electronic technologies impact the surgical patient’s experience to better inform and design a suitable IC process, which is necessary due to current suboptimal IC procedures prevalent across surgical practice [30]. To design the optimal IC process for neurosurgical patients, various patient and non-patient-related factors must be considered. Patient-related factors include age, gender, educational level, previous medical knowledge, and neurocognition. There is evidence that younger patients with greater baseline educational or medical knowledge are more likely to understand information presented via electronic means [82, 92].

The biggest non-patient factor influencing patient satisfaction and understanding is the format in which information is presented. Services must decide which electronic tools should be used, such as animated video explanations, novel interactive or non-interactive multimedia, or simple consent platforms. For objective knowledge gain, the format in which information is presented is crucial, because traditional IC means lengthy documents and brochures with medical terminology that are too advanced for most patients. This process is not inclusive for patients with lower medical literacy, or who are unable to read and digest large amounts of text. Video and animations, on the other hand, are easy to understand and inclusive for all patients. However, it is important to be careful when adopting advanced technologies in a widespread service to ensure that they do not act as an obstacle for patients who are inexperienced with technology, do not have access to technology, or patients with disabilities [35].

Ultimately, it will be a challenge to implement e-consent in low- and middle-income countries (LMIC), with widespread unsuitable infrastructure and different cultural views on medical practice. Different approaches to providing information would be needed, and differences in language, literacy rates, and health education must be considered. The UK (a high-income country (HIC)) is home to a prominent multicultural society that can face its own challenges, such as different attitudes to technology use and health care, language issues, and possibly compliance with reading material. This can lead to insufficient knowledge gain, which could be misinterpreted as inability to understand, rather than because of cultural barriers. Therefore, additional steps must be taken to fully integrate e-consent platforms into LMICs and HICs alike.

When considering vulnerable populations, such as patients with mental health problems, the disabled and the elderly, e-consenting technologies can unintentionally act as an obstacle to understanding. Knowledge gain may become compromised, and this can be misconstrued as a patient not competent to consent.

On the other hand, the greatest advantage of using e-consent is its flexibility, which is useful in situations such as during the COVID-19 pandemic [8], where physical interaction was limited, so information must be communicated effectively and safely via alternative methods. Ensuring that patients receive the relevant information to make a truly informed decision is vital in neurosurgery where patients consent to complex procedures. The reproducibility of e-consent mitigates for time pressures associated with standard verbal IC and has been shown to improve patient comprehension [7].

### IC satisfaction

The results demonstrated that patient satisfaction improved using electronic IC technologies for surgical patients. This was also apparent when used as an adjunct to standard practice (verbal discussion) [2], and during a cross-over study in which participants experienced both processes [91]. Although there are some studies that do not show a significant increase in patient satisfaction after the intervention when examined individually, there is a significant combined effect, as shown by the meta-analysis [1, 9, 10, 16, 20, 23, 27, 42, 46, 63, 69, 71, 75, 78, 82, 86, 87, 89, 90, 92, 95]. Furthermore, as this is a minority of studies, we believe that the non-significant results could be due to various factors, such as patient demographics (baseline understanding, education level, gender), type of surgery, and individual experience with technology. Nevertheless, it is important to consider personal preferences, as some people may be more suited to traditional-personalised consultations with the surgeon. Satisfaction can be subjective, but when used to supplement IC, technology can lead to a greater patient experience. This strengthens patients’ confidence in their healthcare providers and influences treatment adherence, postoperative recovery, and ultimately clinical outcomes.

### Knowledge and understanding

Increased knowledge leads to greater patient autonomy and contributes to a shift towards patient-centred care. In the past, the comprehensiveness of information disclosed to patients was determined by the doctor’s professional judgement. However, the Montgomery ruling [64] challenged this notion and stated that doctors should provide their patients with all relevant information so that they can make an informed decision. This has since influenced British IC practice [50, 80]. For this reason, maximum information provision is crucial to ensure that details (including risks that may or may not be relevant) have been communicated to the patient in a comprehensible and clear way.

In most cases, electronic IC technologies were more effective than standard IC practices in improving patient knowledge, understanding, and retention. However, there were numerous studies that did not show any significant difference [1, 16, 27, 42, 71, 76, 95]. This may be due to small educational differences in patients. As Rossi et al. [75] reported, patients with higher education levels achieved higher test scores. Increased baseline knowledge has previously been shown to increase understanding, which supports the idea that those with higher education, previous surgical experience, or independent research on their surgery would influence the observed results. More complex surgeries would have more content and information to comprehend and retain, posing another challenge to the patient. Therefore, the procedure itself and the volume of information relating to it can influence patient understanding, with more complex surgeries potentially having lower understanding scores. In this case, personal consultations are cardinal, as surgeons can identify concerns and work with the patient sequentially to clarify information. Although digital platforms may not address patient-specific issues if used alone, it was clear in studies where electronic IC technologies were used, in addition to standard practices, that surgeons rated patients in electronic IC intervention arms as more knowledgeable (asked more relevant questions, etc.) [82, 89]. This creates a setting where clinicians must fill gaps in understanding, rather than start from scratch during patient-surgeon discussions, saving time and resources.

Nevertheless, there is an argument to be made about the use of electronic IC technologies instead of the standard oral discussion, as various reports have shown that these interventions performed similar or even better than the input of the healthcare provider [5, 66]. We believe this may be due to the repeatability of electronic IC technologies, which allow patients to re-watch the same information several times until they understand. This cannot be replicated in time-limited surgeon–patient discussions. If electronic methods can match the role of the doctor in preoperative consultations, this would be an invaluable help to replace consent discussions. If used appropriately, digitalization of consent can make health lists more efficiently managed.

### Application to neurosurgery

Ten studies which satisfied inclusion criteria featured neurosurgical patients. These included randomised studies [6, 7, 23, 70], non-randomised studies [51], and single group pretest–posttest design studies [14, 34, 56, 57, 66]. All studies demonstrated either improved patient/surgeon satisfaction and/or patient comprehension with the use of novel information technology adjuncts as part of the consent process. The use of such adjuncts in neurosurgical practice could help standardize and optimize the consent process, ensuring that patients receive the relevant information to make a truly informed decision. The sub-group meta-analysis of these neurosurgical studies outperformed other surgical studies in patient comprehension. This is a testament to the need and usefulness of such a communication platform to exist. In practise, face-to-face clinics are still an important part of the consent process; however, ensuring that patients are not rushed and feel limited by the clinic time is part of our duty to our patients. Electronic consent allows other avenues of communication both visual and audio, to provide the neurosurgical patient with the information they require for the potentially life-changing decision they will make, in the comfort of their own homes. Translation services and multilingual software can have a great impact via electronic consent, to allow patients to read up and understand the risks and benefits of the neurosurgical procedure, which often is complex, that they are about to consent for. It allows the patients to truly understand the pathology, the surgery, and the expected outcome, in their own environment.

### Future directions and recommendations

Research into the use of e-consent provides valuable information to guide future directions to improving the efficiency of surgical consent. It is an important part of the surgical process to ensure patient safety and understanding and to promote patient confidence in the health system, and it has shown promising results so far. Patient satisfaction is generally high through e-consent but can be more effective in certain patient groups or types of operations. Due to differences in literature and variable factors, the most appropriate type of electronic consent technology will vary according to the surgical procedure and the circumstances of the patients. It is important to clarify that the same electronic technology cannot apply to all cases and that individual health services must design their own consenting intervention tailored to their patient’s preferences and resources. This information can be used to improve current practices.

### Limitations

The use of descriptive analysis was a limitation used to review and summarise the advantages, disadvantages, and value of e-consent in surgical practice. The papers included investigated e-consent in elective surgeries, so the results cannot be applied to emergency situations. The studies used various electronic modalities, from videos, computer programmes, and apps to PowerPoint presentations. This is reflected in the high *I*^2^ values for outcomes reporting continuous data in the meta-analysis. The grouping of heterogeneous studies can be attributed to the lack of studies in this area, which itself is the product of the novelty of electronic technologies for information disclosure in clinical practice. Nevertheless, this study cements the substantial value of electronic modalities in IC in surgery and forms the basis for future studies to further investigate this area.

## Conclusions

This is the only meta-analysis regarding the use of electronic consent in surgery and its application to neurosurgery. It has shown both the benefits it withholds and the common usage amongst the worldwide surgical population including neurosurgical cohorts. Alongside streamlining the consent process, e-consent has been shown to improve satisfaction and baseline knowledge in patients undergoing surgical procedures.

### Electronic supplementary material

Below is the link to the electronic supplementary material.

Supplementary file1 (DOCX 31 KB)

## Data Availability

The data used to support the findings of this study are included in the article tables. Raw data files can be accessed upon request to corresponding author.

## Abbreviations

CDs: Compact discs
DEF: Data extraction form
HIC: High-income country
IC: Informed consent
LMIC: Low- and middle-income countries
MCQs: Multiple-choice questions
NHLBI: National Heart, Lung, and Blood Institute
NSRIs: Non-randomised studies of healthcare interventions
PPD: Pre-post design
PRISMA: Preferred Reporting Items for Systematic Reviews and Meta-Analyses
RCTs: Randomised controlled trials
SM: Supplementary materials
SMD: Standardised mean difference
