# Supplementary material for: The use of e-consent in surgery and application to neurosurgery: a systematic review and meta-analysis
Source: Acta Neurochir (Wien). 2023 Sep 11;165(11):3149–80. doi: 10.1007/s00701-023-05776-3 (PMC10624752; doi:10.1007/s00701-023-05776-3)
Supplement: Supplementary file 1 — Supplementary file1 (DOCX 31 KB) [file 701_2023_5776_MOESM1_ESM.docx]

**Supplemental Material**

| Search criteria | Search terms | Number of results |
| --- | --- | --- |
| 1 | **surgery.mp.** | **6,877,038** |
| 2 | **surgical patient.mp.** | **58,750** |
| 3 | **surgical case.mp.** | **3,649** |
| 4 | **surgical procedure.mp.** | **120,810** |
| 5 | **surgical operation.mp.** | **11,066** |
| 6 | **surgical specialties.mp.** | **6,684** |
| 7 | **1 or 2 or 3 or 4 or 5 or 6** | **6,920,748** |
| 8 | **consent.mp.** | **318,103** |
| 9 | **informed consent.mp.** | **229,390** |
| 10 | **informed consent process.mp.** | **4,297** |
| 11 | **information provision.mp.** | **5,318** |
| 12 | **informed decision.mp.** | **12,695** |
| 13 | **consent forms.mp.** | **6,230** |
| 14 | **8 or 9 or 10 or 11 or 12 or 13** | **334,258** |
| 15 | **video.mp.** | **384,851** |
| 16 | **computer.mp.** | **2,580,734** |
| 17 | **computer-based techniq*.mp.** | **300** |
| 18 | **e-consent.mp.** | **128** |
| 19 | **electronic consent.mp.** | **250** |
| 20 | **multimedia.mp.** | **22,523** |
| 21 | **multi-media.mp.** | **1,673** |
| 22 | **multimedia tool*.mp.** | **380** |
| 23 | **Multi-media tool*.mp.** | **18** |
| 24 | **digital.mp.** | **438,626** |
| 25 | **digital instrument.mp.** | **87** |
| 26 | **Digital technolog*.mp.** | **12,698** |
| 27 | **digital tool*.mp.** | **3,062** |
| 28 | **digital intervention.mp.** | **986** |
| 29 | **audio-video.mp.** | **1,861** |
| 30 | **video-only.mp.** | **329** |
| 31 | **interactive multimedia.mp.** | **1,188** |
| 32 | **interactive multi-media.mp.** | **43** |
| 33 | **computer graphic*.mp.** | **29,404** |
| 34 | **audiovisual aid*.mp.** | **9,985** |
| 35 | **audio-visual.mp.** | **7,306** |
| 36 | **smartphone.mp.** | **47,889** |
| 37 | **mobile application*.mp.** | **33,725** |
| 38 | **telemedicine.mp.** | **95,927** |
| 39 | **online system*.mp.** | **39,067** |
| 40 | **videotape recording*.mp.** | **15,737** |
| 41 | **15 or 16 or 17 or 18 or 19 or 20 or 21 or 22 or 23 or 24 or 25 or 26 or 27 or 28 or 29 or 30 or 31 or 32 or 33 or 34 or 35 or 36 or 37 or 38 or 39 or 40** | **3,412,315** |
| 42 | **7 and 14 and 41** | **5,635** |
| 43 | **remove duplicates from 42** | **4,744** |
| 44 | **patient satisfaction.mp.** | **289,331** |
| 45 | **patient understanding.mp.** | **4,382** |
| 46 | **patient comprehension.mp.** | **996** |
| 47 | **patient knowledge.mp.** | **6,223** |
| 48 | **participation.mp.** | **608,248** |
| 49 | **patient participation.mp.** | **69,389** |
| 50 | **satisfaction.mp.** | **718,315** |
| 51 | **understanding.mp.** | **2,725,641** |
| 52 | **comprehension.mp.** | **156,650** |
| 53 | **pre-operative anxiety.mp.** | **471** |
| 54 | **information recall.mp.** | **758** |
| 55 | **information retention.mp.** | **600** |
| 56 | **44 or 45 or 46 or 47 or 48 or 49 or 50 or 51 or 52 or 53 or 54 or 55** | **4,049,099** |
| 57 | **42 and 56** | **745** |
| 58 | **remove duplicates from 57** | **579** |

**Table A: Search terms used for: Ovid® Medline ALL; APA Psycinfo; Global Health; and Embase.**
